# The cellular senescence of leukemia-initiating cells from acute lymphoblastic leukemia is postponed by *β*-Arrestin1 binding with P300-Sp1 to regulate hTERT transcription

**DOI:** 10.1038/cddis.2017.164

**Published:** 2017-04-20

**Authors:** Shan Liu, Haiyan Liu, Ru Qin, Yi Shu, Zhidai Liu, Penghui Zhang, Caiwen Duan, Dengli Hong, Jie Yu, Lin Zou

**Affiliations:** 1 Center for Clinical Molecular Medicine, Children's Hospital, Chongqing Medical Universtiy, Chongqing 400014, China; 2 Ministry of Education Key Laboratory of Child Development and Disorders, Chongqing 400014, China; 3 Key Laboratory of Pediatrics in Chongqing, Chongqing 400014, China; 4 Division of Hematology, Children’s Hospital, Chongqing Medical University, Chongqing 400014, China; 5 Center for Clinical Laboratory Medicine, Children's Hospital, Chongqing Medical Universtiy, Chongqing 400014, China; 6 Key Laboratory of Cell Differentiation and Apoptosis, Shanghai Jiao Tong University School of Medicine, Shanghai 200025, China; 7 Chongqing Stem Cell Therapy Engineering Technical Research Center, Chongqing 400014, China

## Abstract

Although we previously reported that the self-renewal of leukemia-initiating cells of B-lineage acute lymphoblastic leukemia (B-ALL LICs) was regulated by *β*-Arrestin1, a multiple-function protein, the cellular senescence is critical for LICs fate and leukemia progress, and worthy for further investigation. Here we found that depletion of *β-Arrestin1* extended the population doubling time and the percentage of senile cells, the signatures of cellular senescence, of B-ALL LICs. Moreover, lack of *β-Arrestin1* enhanced the expression of proteins (CBX, HIRA) and genes (*P53*, *P16*) related to senescence in leukemic Reh cells and B-ALL-LICs-derived leukemic mice. Further results showed that loss of *β-Arrestin1* induced senescence of Reh cells through mediating *hTERT*-telomerase-telomere axis, which was reversed by BIBR1532, the telomerase activity inhibitor. Importantly, depletion of *β-Arrestin1* decreased the binding of Sp1 to *hTERT* promoter at the region of −28 to −36 bp. The anti-sense oligonucleotide of this key region downregulated the transcription of *hTERT* and aggravated the senescence of Reh cells. Further data demonstrated that the depleted *β-Arrestin1* reduced the interaction of P300 with Sp1, thus to reduce Sp1 binding to *hTERT* promoter, downregulate *hTERT* transcription, decrease telomerase activity, shorten telomere length, and promote Reh cell senescence. Interestingly, the percentage of senile cells in B-ALL LICs was decreased, which was negatively correlated to good prognosis and *β-Arrestin1* mRNA expression in childhood B-ALL patients. Our study shed a light on the senescence of B-ALL LICs and is regulated by *β-Arrestin1*, providing the potential therapeutic target of leukemia by promoting cellular senescence with a key region of *hTERT* promoter.

Acute lymphoblastic leukemia (ALL) is the most common tumor in children under age 15. According to the affected cells, ALL is divided into B-lineage acute lymphoblastic leukemia (B-ALL) and T-lineage acute lymphoblastic leukemia (T-ALL). The long-term rates of event-free survival (EFS) for childhood B-ALL have approached close to 90%, from <10% in the 1960s, in developed countries.^1, 2^ However, about 10–15% of relapse and refractory B-ALL patients have still lower overall survival (OS) and EFS rates.^2^ The exact mechanism of relapse and refractory B-ALL is unclear. In recent years, leukemia-initiating cells (LICs), the cell population with the self-renewal capacity to initiate and maintain leukemia, have been found pivotal in relapse and drug resistance for B-ALL because of the properties LICs that share with normal hematopoietic stem cells (HSCs) such as the immunophenotyping (CD34^+^CD38^−^CD19^+^) and maintenance of a quiescent state that makes the cells unresponsive to cell cycle-specific cytotoxic agents.^3^ Besides the self-renewal ability of LICs, the cellular senescence of LICs is a critical factor for the leukemia progression,^4^ and aroused great concerns in researchers.

The cellular senescence means a terminal growth arrest, which includes premature senescence and replicative senescence. Premature senescence, mainly induced by stress, oncogenes, and tumor suppressors,^5^ has been increasingly demonstrated to be critical for the development of several kinds of leukemia.^6^ Replicative senescence is also called telomere-induced senescence, primarily due to shortened telomere, and the senescence is present in Ph^+^ CML^7^ and chronic lymphocytic leukemia (CLL).^8^ Most of the human cancers have acquired mechanisms to maintain telomeres, generally through high expression of telomerase. Telomere-induced senescence also has been shown to act as a tumor suppressor in telomerase-deficient mice.^9^ Therefore, telomere and telomerase are keys for cellular senescence and tumorigenesis.

Human telomerase reverse transcriptase (hTERT) is one of three telomerase main components, together with the human telomerase RNA molecules (hTR) and telomerase-associated proteins (TAP), which determines the rate of telomerase activity and expresses in most malignant tumors but not in normal tissues.^10, 11^ High *hTERT* expression was observed in some subtypes of leukemia like CLL and T-ALL.^12, 13^ The expression of *hTERT* gene is governed by its transcription through its promoter, and the transcription factor is the main regulatory aspect.^14, 15^ Some transcription factor-binding sites are in the region of the *hTERT* promoter, including Sp1, c-Myc, USF, and so on.^14, 15^ The Sp1 composite element centered from −1 to −110bp and with five binding sites in the proximal of *hTERT* promoter is particularly crucial for basal *hTERT* expression.^14^ Sp1 was identified as an activator for *hTERT* transcription in some tumors, including those of primary effusion lymphoma,^16^ prostate cancer^17^ and even Jurkat T cells.^18^ Sp1 could combine with factors like c-Myc,^14^ Sp3 (ref.^18^ to promote *hTERT* transcription, which also needs a permissive chromatin environment.^19^ For example, P300, a histone acetyltransferase, could not only bind with Sp1 (ref.^20^ but also be involved in the chromatin remodeling.^21^ Whether Sp1 binding with P300 mediates *hTERT* transcription and the *hTERT*-telomerase-telomere axis in B-ALL replicative senescence needs exploration.

*β-Arrestin1*, the important scaffold protein of *β-Arrestin1* family, is ubiquitously distributed and of more concern regarding cancer progression, which transduce signals through and regulate the PI3K/AKT, Wnt, and Hedgehog signaling pathways to mediate cell development and differentiation, associated with the progression of malignancies.^22^ Both *β-Arrestin1* and *β-Arrestin2* could mediate the initiation and maintenance of myeloid leukemia.^23, 24^ In particular, *β-Arrestin1* could regulate histone proteins' modification and gene transcription by coupling with CREB and YY1 to further regulate cell function.^23, 24^ Our previous studies showed that overexpression of *β-Arrestin1* was associated with a high risk of pediatric B-ALL and promoted the self-renewal of B-ALL LICs.^25, 26^ Given that the cellular senescence of LICs is essential for B-ALL progress, we are interested to further explore the critical role of *β-Arrestin1* in the cellular senescence of LICs and B-ALL progress.

Our data revealed that depletion of *β-Arrestin1* facilitated cell senescence of B-ALL LICs *in vivo* and *in vitro*, by regulating *hTERT* transcription through inducing P300-Sp1 interaction at −28 to −36 bp of *hTERT* promoter, which was further illustrated by the data from clinical samples that decreased senile cells and elevated expression of *β-Arrestin1* predicted poor prognosis in B-ALL, providing the potential therapeutic target of leukemia by promoting cellular senescence.

## Results

### Loss of *β-Arrestin1* accelerated senescence in B-ALL LICs

**Figure 1:**
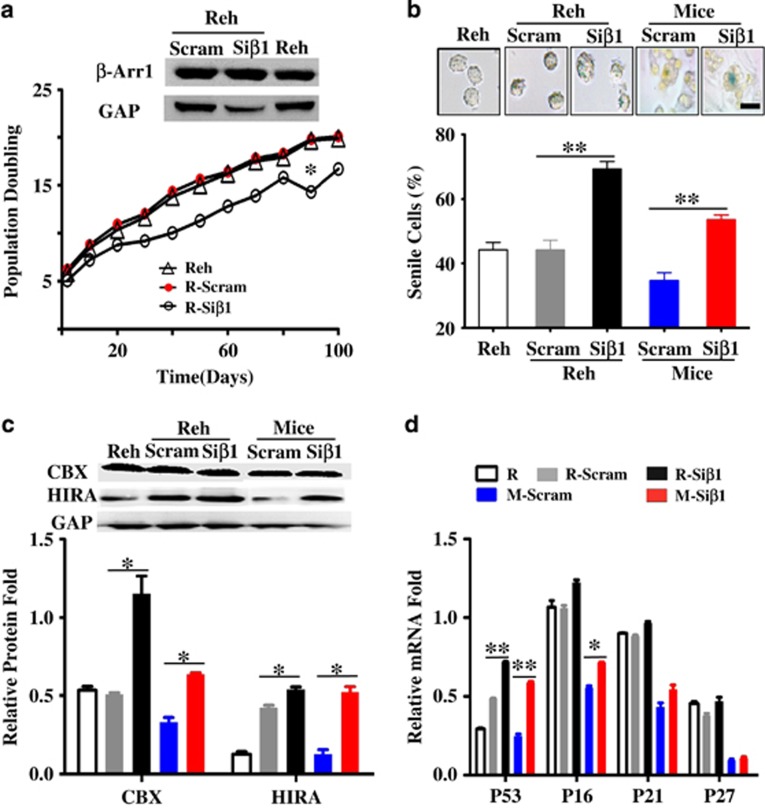
Loss of *β-Arrestin1* promotes the senescence in B-ALL LICs. Reh cells and isolated CD34^+^CD38^−^CD19^+^ cells (LICs) were transiently infected with Si-*β-Arrestin1* (Si*β*1) or scrambled shRNA vector (Scram) lentivirus particles, named R-Si*β*1, R-Scram, LICs-Si*β*1, and LICs-Scram cells. 1 × 10^4^ LICs-Si*β*1 or LICs-Scram cells were injected intravenously into irradiated NSG mice (*n*=10), named M-Si*β*1 and M-Scram mice. The mice were dead or killed 96 days after injecting cells with obvious leukemia symptoms. BM cells, PB cells, and tissues were collected. (**a**) R-Si*β*1, R-Scram and Reh were serially subcultured in an RPMI1640 medium, and the times of doubling populations were compared. The difference between R-Si*β*1 and R-Scram was performed. (**b**) The representative images of SA-*β*-gal staining (top) and the statistical data (bottom) were shown *in vitro* and *in vivo*. (**c**)Total protein was purified from R-Si*β*1, R-Scram, and PB cells of M-Si*β*1 or M-Scram mice. The expression of HIRA and CBX, the marker for senescence, was detected by Western Blot. Representative western blot images (top) and the levels of HIRA and CBX were quantified relative to their respective GAPDH level (bottom). (**d**) RNA was extracted from R-Si*β*1, R-Scram, and PB cells of M-Si*β*1 or M-Scram mice. The expression of *P53, P16, P21*, and *P27*, which is related with senescence, was detected by real-time PCR. The levels of genes were quantified relative to their respective *GAPDH* level. Differences among groups were calculated by *t*-test. *P<0.05, **P<0.001; GAP, inner control *GAPDH* gene; M, NSG mice; *n*, number; OS, overall survival; R, Reh cells;. Scale bar, 5 *μ*m

On the basis of our previous report that *β-Arrestin1* regulated the self-renewal of B-ALL LICs^5^ and the cellular senescence is another critical factor for LICs and leukemia progress;^4^ here we further investigate whether the cellular senescence of B-ALL LICs was mediated by *β-Arrestin1*. Beside isolation and identification of LICs from B-ALL as our latest report,^26^ we screened *β-Arrestin1* expression and senescence status in different B-ALL cells to obtain the qualified cell models for B-ALL LICs. The results showed that only progenitor Reh cells had the similar pattern of cellular senescence and *β-Arrestin1* expression with that of the LICs from B-ALL patients. We adopted Reh cells as the B-ALL LICs model for further exploration, and then infected Reh cells and leukemic mice injected the LICs with Si-*β-Arrestin1* (Si*β*1) lentivirus particles,^26^ named Reh-Si*β*1 or Mice-Si*β*1 (Supplementary Figure S2). The expression of *β-Arrestin1* was then detected in Reh-Si*β*1, Reh-Scram (Supplementary Figure S6D), Mice-Scram and Mice-Si*β*1 (Supplementary Figure S2D) by western blot. The impaired expression of *β-Arrestin1* extended the survival time of leukemic mice by Kaplan–Meier analysis (Supplementary Figure S2F). Reh-Si*β*1 cells had lower population doubling times than that of Reh-Scram control cells through serial subculture (Figure 1a). Moreover, more senile cells were observed in Reh-Si*β*1 cells and BM cells from Mice-Si*β*1 (Figure 1b). The protein markers for senescence,^5^ CBX and HIRA, were then measured by western blot, and the results demonstrated that both the expression of CBX and HIRA were higher in Reh-Si*β*1 cells and BM cells from Mice-Si*β*1 (Figure 1c). Further, the expression of senescence-associated genes, including *P16, P53, P21,* and *P27* were detected by real-time RT-PCR, and the data showed that the expression of these genes was higher in Reh-Si*β*1 cells and BM cells from Mice-Si*β*1 (Figure 1d) than those from Reh-Scram cells and BM cells from Mice-Scram, respectively. Taken together, those observations suggest that the cellular senescence of B-ALL LICs is promoted by Si-*β-Arrestin1*.

### Loss of *β-Arrestin1* accelerated senescence *via* telomerase and telomere

To elucidate the mechanism of *β*-Arrestin1 regulating the senescence of B-ALL LICs, we analyzed the reactive oxygen species (ROS) for premature senescence and the telomere length for replicative senescence. The ROS level was labeled with DCFH and observed by fluorescence spectroscopy in Reh-Si*β*1 cells, Reh-Scram cells, and BM cells from Mice-Si*β*1 or Mice-Scram, but there was no significant difference among these groups (Supplementary Figure S4). Hence, we focused on the telomerase and telomere.

**Figure 2:**
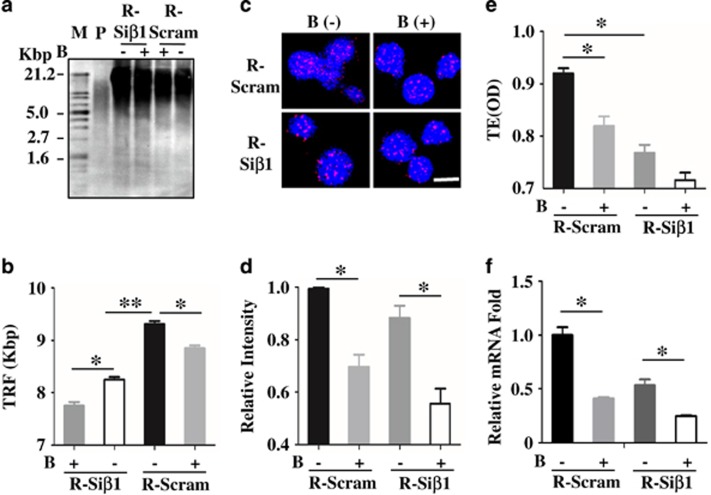
Loss of *β-Arrestin1* inhibits *hTERT* expression, decreases telomerase activity, and shortens telomere length in B-ALL LICs. DNA was extracted from R-Si*β*1 cells, R-Scram cells. The length of telomere was detected by Southern blot. Calculating telomere length was performed with optical density analysis, according to the kit’s protocol. The average length of telomere (TRF) in different groups was calculated and compared with the control group. The representative images of the Southern blot (**a**) and the TRF (**b**) in Reh cells. The length of telomere was also detected by FISH in smear. The representative images of FISH (**c**); After calculating the fluorescence intensity of telomeres, the value was quantified relative to the level of Scram group without BIBR1532 and are presented as the relative intensity of fluorescence (bottom) in Reh cells (**d**). The activity of telomerase was measured by PCR-ELISA in Reh cells (**e**). The *hTERT* mRNA expression fold was detected by RT-PCR in Reh cells (**f**). All *P*-values were calculated by *t*-test. *P<0.05, **P<0.001; B, the inhibitor of telomerase (BIBR1532); M, marker; P, positive control; TE, the activity of telomerase; TRF, the average length of telomere. Scale bar, 10 *μ*m

To further verify the role of *β-Arrestin1* on telomere and telomerase in B-ALL LICs, we measured the telomere length, telomerase activity, and *hTERT* expression in Reh-Si*β*1, Mice-Si*β*1, and their control groups. The shortened telomere length, decreased telomerase activity, and reduced *hTERT* mRNA expression were observed in Reh-Si*β*1 (Figures 2a, c, e, and f) and Mice-Si*β*1 (Supplementary Figures S7A, S7C, S7E, and S7F) correspondingly. Moreover, BIBR1532, the inhibitor of telomerase activity, was applied to explore the specificity of telomere and telomerase in the process of *β-Arrestin1* regulating the senescence of LICs, after successfully screening its best dosage (3 *μ*mol/l), time (48 h) (Supplementary Figure S5A) and *hTERT* mRNA expression in Reh cells (Supplementary Figure S5B), and *hTERT* mRNA expression in Reh-Si*β*1 and Reh-Scram (Supplementary Figure S5C). The results showed that BIBR1532 could inhibit *hTERT* mRNA expression and telomerase activity, with weak inhibition of telomere length (Figure 2 and Supplementary Figure S7). These results together demonstrated that *β*-Arrestin1 might regulate the senescence of B-ALL LICs through *hTERT*-telomerase-telomere axis.

### Loss of *β-Arrestin1* reduced *hTERT* transcription by binding with Sp1

**Figure 3:**
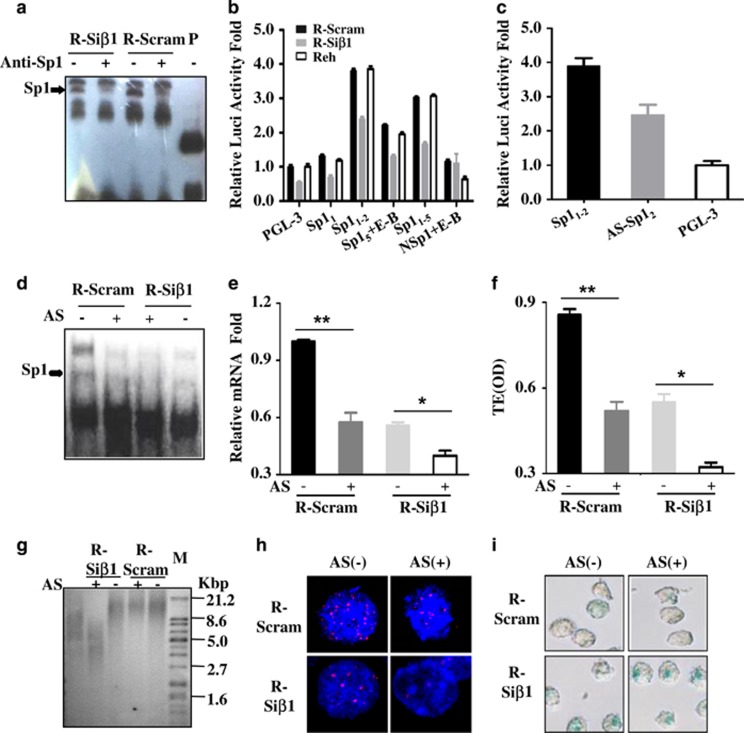
Loss of *β-Arrestin1* reduces the transcription of *hTERT*. (**a**) Different Reh cells were collected. We performed supershift by EMSA on purified nucleus protein to detect Sp1 binding with the hTERT promoter. (**b**) Reh cells were transfected by different plasmids with various cloned fragments. The luciferase activity was detected by dual-luciferase reporter assay and normalized by Renilla luciferase. The relative luciferase activity was viewed as 1.0 in Reh with a PGL-3-basic vector. (**c**) The anti-sense oligonucleotide was designed for the binding sites (−28 to −36 bp) of the hTERT promoter, measuring the relative luciferase activity, and comparing between Reh with or without the anti-sense oligonucleotide. R-Si*β*1 cells and R-Scram cells were transfected by anti-sense oligonucleotide of Sp1_2_ (−28 to −36 bp). The cells' total DNA and protein were collected. Then we detected the binding of the *hTERT* promoter with Sp1 by EMSA (**d**) measured the relative *hTERT* mRNA fold by RT-PCR (**e**) detected the activity of telomerase by PCR-ELISA (**f**) measured the length of telomere by Southern blot (**g**) and FISH (**h**), respectively, and obtained representative images through SA-*β*-gal staining (**i**). Anti-Sp1, antibody for Sp1; AS, anti-sense oligonucleotide. AS, anti-sense oligonucleotide of Sp1_2_ (−28 to −36 bp); Ctrl, Reh cells not transfected with anti-sense oligonucleotide of Sp1_2_ (−28 to −36 bp); E-B, E-box region; NSP, the fragment excluding the binding sites for Sp1; P-C, positive control; TE, the activity of telomerase; TRF, the average length of telomere. Scale bar, 10 *μ*m

In the light of the activity of telomerase, which is positively correlated with the length of the telomere, is known to be rate-limiting regulated by *hTERT* gene.^11^ Sp1 is a transcription activator to stimulate *hTERT* transcription and there are five Sp1-binding sites in the proximal region of the *hTERT* promoter^14^ (Supplementary Figure S6A). Considering that the *hTERT* expression is governed by its transcription through its promoter, we illustrated that *β-Arrestin1* didn’t affect the expression of Sp1 protein in nuclear (Supplementary Figure S6B), but *β-Arrestin1* increased the binding of Sp1 to *hTERT* promoter by electrophoretic mobility shift assay (EMSA) in Reh cells (Figure 3a). Then, we cloned different binding fragments for Sp1 in *hTERT* promoter (Supplementary Figure S6C) into the PGL-3 basic reporter vector to find the most important site for *hTERT* transcription, and then transfected different fragments of *hTERT* reporter vectors with various Sp1-binding sites into Reh-Si*β*1 and Reh-Scram cells. The results from luciferase reporter assay showed that luciferase was less active in Reh-Si*β*1 cells than that in Reh-Scram cells, and the highest activity was detected in Reh cells transfected with the second binding fragment for Sp1 (Sp1_2_) (− 28 to −36 bp) (Figure 3b).

To study the specificity of binding sites Sp1_2_ in affecting the transcription of the *hTERT* gene, we designed anti-sense oligo-DNA fragments for Sp1_2_ (AS-Sp1_2_) and transfected it into Reh cells. The data showed less luciferase activity in Reh cells transfected with AS-Sp1_2_ than in those without transfection (Figure 3c). Subsequently, in Reh cells transfected with AS-Sp1_2_, the binding of Sp1 to *hTERT* promoter was decreased, still measured by EMSA (Figure 3d). In addition, the reduced *hTERT* expression (Figure 3e), the declined telomerase activity (Figure 3f), and the shortened telomere length were consistently observed in Reh cells transfected with AS-Sp1_2_ as well (Figures 3g and h, the statistical graphs were in Supplementary Figures S8A and S8B, respectively). Moreover, there were more senile cells than those in Reh cells without AS-Sp1_2_ transfection (Figure 3i, statistical graphs were in Supplementary Figure S8C). These data indicated that the region (−28bp to −36bp) in the *hTERT* promoter was the core region for Sp1 binding, which further stimulated *hTERT* transcription and senescence regulated by *β*-Arrestin1 in B-ALL Reh cells.

### Loss of *β-Arrestin1* reduced Sp1 interacting with P300 in *hTERT* promoter

**Figure 4:**
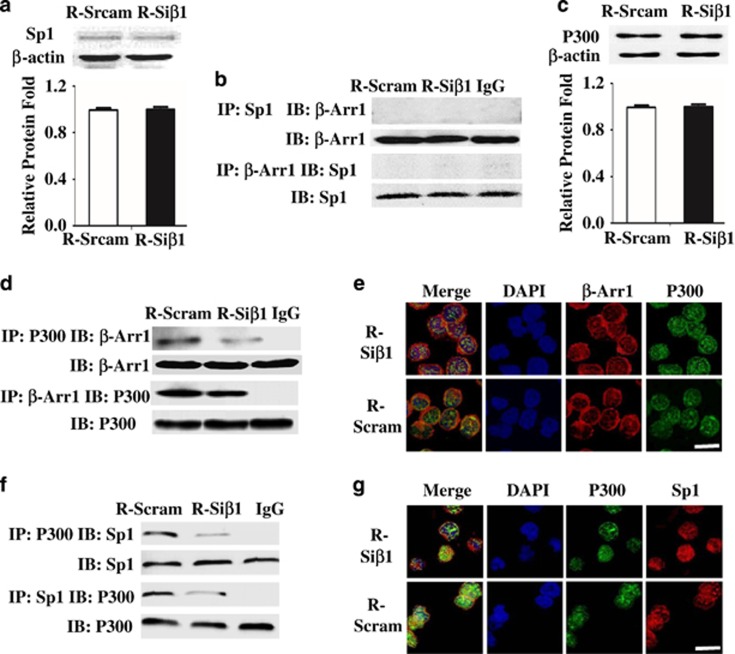
Loss of *β-Arrestin1* decreases the formation of P300-Sp1 complex. Protein was extracted from R-Scram cells and R-Si*β*1 cells, and smears were prepared. (**a**) Representative western blot images of Sp1 protein (top) and the levels of Sp1 protein were quantified relative to those of *β*-actin and are presented as folds compared with the control cells (bottom). (**b**) The colocalization of *β-Arrestin1* and Sp1 by Co-IP. (**c**) Representative western blot images of P300 protein (top) and the levels of P300 protein were quantified relative to those of *β*-actin and are presented as folds compared with the control cells (bottom). (**d**) The colocalization of *β*-Arrestin1 and P300 by Co-IP. (**e**) The binding of *β-Arrestin1* and P300 by immunofluorescence and confocal microscopy. (**f**) The colocalization of P300 and Sp1 by Co-IP. (**g**) The binding of P300 and Sp1 by immunofluorescence and confocal microscopy. Scale bar, 5 *μ*m

To further find how *β-Arrestin1* regulates Sp1 to bind with *hTERT* promoter, we first speculated that Sp1 is regulated by *β-Arrestin1* directly. However, neither the expression of Sp1 (Figure 4a) by western blot nor the binding of *β-Arrestin1* with Sp1 (Figure 4b) by Co-IP was altered in Reh cells loss of *β*-Arrestin1.

We then assumed that *β-Arrestin1* could help form a protein–protein complex to bind with *hTERT* promoter. In view of Sp1 is reported to work with P300 in activating gene transcription,^20^ we further analyzed the role of P300 in this process. Our data showed that Reh cells with *β-Arrestin1*-Arrestin1 depletion were not the reduced expression of total P300 protein (Figure 4c), but the loss of *β-Arrestin1* reduced the interacting of *β*-Arrestin1 with P300 as shown by Co-IP (Figure 4d) and immunofluorescence confocal assay (Figure 4e), and the binding of Sp1 with P300 as shown by Co-IP (Figure 4f) and immunofluorescence confocal assay (Figure 4g). These findings suggest that P300, as the important mediator, could promote the formation of *β*-Arrestin1-P300-Sp1 complex, and that the depletion of *β-Arrestin1* might decrease this complex formation.

**Figure 5:**
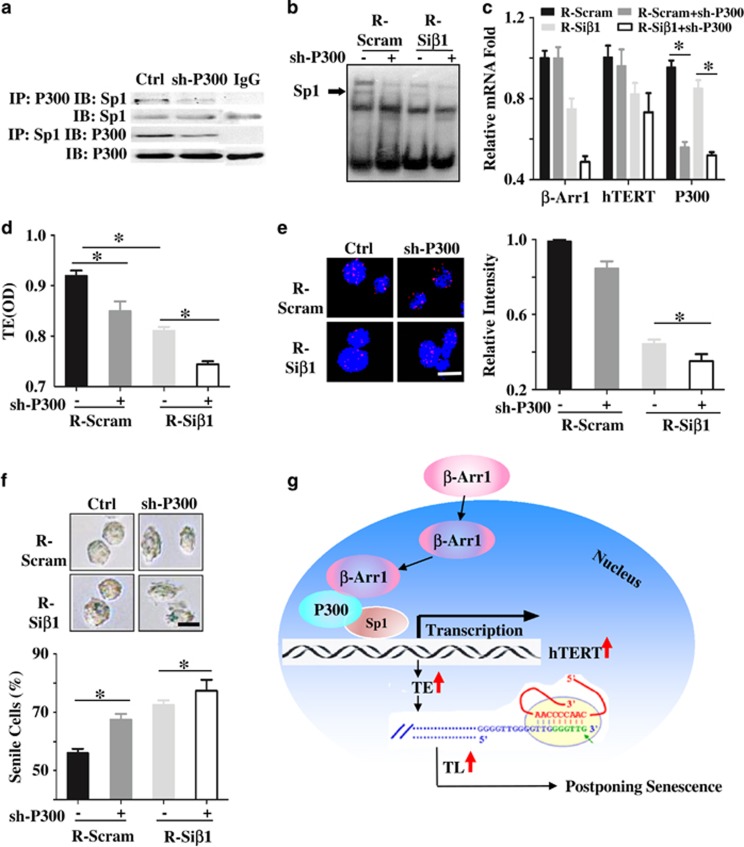
P300 is necessary for *β-Arrestin1* regulating the senescence via the *hTERT*-telomerase-telomere axis in B-ALL. (**a**) Reh cells were transfected with shRNA-*P300*, and collected the cells, and then measured the binding of P300 with Sp1 by Co-IP. Then, R-Scram cells and R-Si*β*1 cells were transfected with shRNA-p300. The binding of the hTERT promoter with Sp1 by EMSA (**b**), the relative hTERT mRNA fold by RT-PCR (**c**), the activity of telomerase by PCR-ELISA (**d**), the length of telomere by FISH (**e**), and the senescence by SA-*β*-gal staining (**f**) were shown. The scheme of this study, which showed that *β*-Arrestin1 enhances the P300-Sp1 complex and the binding of the complex to the hTERT promoter, thus regulating the senescence of B-ALL via the *hTERT*-telomerase-telomere axis (**g**). *β*-Arr 1, the gene of *β*-arrestin1; Ctrl, transfected without oligonucleotide shRNA-*P300*; sh-*P300*, transfected with oligonucleotide shRNA-*P300*; TE, telomerase activity; TL, telomere length. Scale bar, 5 *μ*m

To further testify the hypothesis, we chemically synthesized small RNA interference strands for *P300* (sh-P300). Four sh-P300s targeting different fragments of the *P300* gene were designed, named sh477-1, sh478-1, sh479-1, and sh480-1 (sequences of sh-*P300s* were listed in Supplementary Table S2). The expression of *P300* was detected in Reh cells transfected with these fragments, and the sh479-1 fragment with the best inhibition was used for further research (data not shown). We found that the depletion of *P300* reduced the binding of P300 with Sp1 (Figure 5a) and the binding of P300-Sp1 complex to *hTERT* promoter (Figure 5b). Then, the decreased *hTERT* transcription (Figure 5c) and the declined telomerase activity (Figure 5d), the shortened telomere length (Figure 5e), and the enhanced percentage of senile cells (Figure 5f) were observed in Reh cells knocked-down P300. Altogether, these results show that *β-Arrestin1* increases the P300-Sp1 complex and the binding of P300-Sp1 to *hTERT* promoter, upregulates *hTERT* transcription, increases telomerase activity, and extends telomere length, thus, postponing cell senescence and changing the cell fate of B-ALL LICs (Figure 5g).

### The percentage of senile cells was negatively correlated with *β*-Arrestin1 expression and poor prognosis in LICs from B-ALL patients

**Figure 6:**
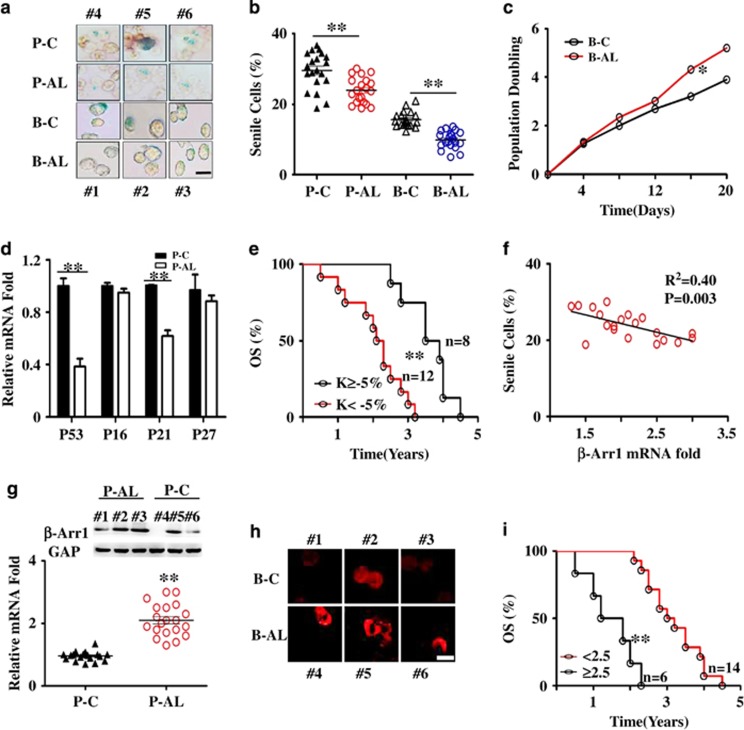
Less senile cells are correlated with high expression of *β-Arrestin1* in B-ALL. BM cells and PB cells were collected from 20 B-ALL patients (P-AL from PB cells, B-AL from BM cells) and 20 non-leukemic patients as a control (P-C from PB cells, B-C from BM cells). BM smears were prepared. RNA and protein were purified from PB cells. The *β-Arrestin1*, *P53, P16, P21* and *P27* mRNA were detected by real-time RT-PCR, and the data are presented as folds using *GAPDH* as the reference gene, and the relative *β-Arrestin1/GAPDH* expression in the P-C was viewed as 1.0-fold. (**a**) PB cells and BM cells were stained by SA-*β*-gal, the stained cells were prepared on smear. The representative images were shown. Both P-C and B-C were from patients #4, #5, #6; both P-AL and B-AL were from person No. 1, No. 2, No. 3. (**b**) The statistical graphs for SA-*β*-gal staining of all specimens. The data are represented as the means±S.E.M and *P*-value is calculated by *t*-test. (**c**) B-C and B-AL cells were serially cultured in an RPMI1640 medium, and the times of doubling populations were compared by *t*-test. (**d**) The expression of *P53, P21, P16* and *P27* mRNA were detected by real-time RT-PCR. The statistics for all specimens were shown with the means±SEM and *P*-value is calculated by *t*-test. (**e**) Overall survival of B-ALL based on the K value (k value=the percentage of senile cells in P-AL cells subtract the average percentage of senile cells in P-C cells), was analyzed by the Kaplan–Meier method, with K=−5% as the cutoff value in P-AL cells. (**f**) The correlation analysis of the percentage of senile cells with mRNA folds of *β-Arrestin1* gene. (**g**) Representative western blot images of *β*-Arrestin1 (top) and the relative mRNA expression of *β-Arrestin1* (bottom) in patients. (**h**) Representative immunofluorescence images of *β*-Arrestin1 expression in patients. (**i**) the overall survival of B-ALL based on *β-Arrestin1* mRNA expression was analyzed by Kaplan–Meier method, with 2.5-fold *β-Arrestin1* mRNA expression as the cutoff value. *P<0.05, **P<0.001; P-C, PB cells in non-leukemic patients; P-AL, PB cells in B-ALL patients; B-C, BM cells in non-leukemic patients; B-AL, BM cells in B-ALL patients; *β*-Arr 1, the gene of *β*-arrestin1; GAP, inner control GAPDH gene; *n*, the number of patients; OS, overall survival; Scale bar, 5 *μ*m

To further analyze the clinical significance of the cellular senescence of B-ALL LICs, we collected bone marrow from B-ALL patients, to isolate LICs to investigate the percentage of senile cells and the expression of *β-Arrestin1* simultaneously, and further to investigate the survival time of these patients. The data showed that the ratio of senile cells is less (9 *versus* 15% in BM; 22 *versus* 30% in PB) (Figures 6a and b) through SA-*β*-gal staining and the population doubling time was shortened through serial subculture in B-ALL compared to their respective controls (Figure 6c). Moreover, the expression of senescence-related genes was lower in B-ALL patients than those from controls, especially the expression of *P53* and *P16* (Figure 6d).

Overall survival (OS) of B-ALL based on the *K*-value (*K*=percentage of senile cells in PB-ALL (P-AL) cells subtract the average percentage of senile cells in PB-Control (P-C) cells), was analyzed by the Kaplan–Meier method. *K*<−5% could distinguish B-ALL patients' with a poor prognosis from those with a good prognosis (Figure 6e), suggesting that a low percentage of senile cells in PB cells were positively associated with an unfavorable prognosis in B-ALL.

On the basis of our previous studies,^25, 26^ here we further investigated whether *β-Arrestin1* affected the OS of leukemia patients with detailed listed information (Supplementary Table S1). Enhanced expression of *β-Arrestin1* was observed (Figures 6g and h) and the median value of *β*-Arrestin1 mRNA was about 2.2-fold in B-ALL patients compared with corresponding controls. We then chose 2.5-fold as a cutoff value for the survival analysis. Leukemic patients (Figure 6i) with ≥2.5-fold of *β-Arrestin1* mRNA expression level had poor prognosis (patients: 2.8 years *versus* 3.9 years), indicating that a high expression of *β*-Arrestin1 is positively correlated with an unfavorable prognosis for B-ALL patients. Interestingly, we found that the mRNA expression of *β*-Arrestin1 was negatively correlated with senescence in B-ALL patients (*P*<0.001, R^2^=0.55) (Figure 6f).

In order to further investigate the relationship of senescence with *β*-Arrestin1 on LICs, we isolated different fractions of LICs from BM cells of 20 newly diagnosed pediatric B-ALL patients, using MACS and identified them using FACS (Supplementary Figure S1A). The least senile cells were observed on the LICs (Supplementary Figures S1B and S1C), and the highest expression of *β-Arrestin1*-Arrestin1 was also demonstrated on the LICs, as shown in our latest report.^26^ All four fractions showed that a smaller percentage of senile cells was correlated with higher expression of *β-Arrestin1*, but significant correlation was observed only in LICs (*P*<0.001, *R*^2^=0.91) (Supplementary Figures S1D–S1G).

After isolation and identification, the LICs and other fractions as control from newly diagnosed pediatric B-ALL patients were injected into mice, respectively, we then collected PB and BM specimens after identifying leukemia (Supplementary Figure S2), and found that less senile cells (Supplementary Figure S3D) and higher expression of *β-Arrestin1* were shown in leukemic mice derived from LICs fraction (Supplementary Figures S3A and S3B). To explore the relationship of survival with senescence and the expression of *β-Arrestin1* in leukemic mice, we got the similar survival results with leukemic patients (Supplementary Figures S3E and S3C). The mRNA expression of *β-Arrestin1* was also negatively correlated with senescence in the leukemic mice (*P*<0.001, R^2^=0.67) (Supplementary Figure S3F). Altogether, our data indicate that the role of LICs’ senescence of related to *β-Arrestin1* on predictive ability for B-ALL.

## Discussion

Both cellular senescence and *β-Arrestin1* play critical roles in the survival of pediatric B-ALL patients. Here we uncovered that depletion of *β*-Arrestin1 enhanced cell senescence of B-ALL LICs *in vivo* and *in vitro* by regulating the *hTERT*-telomerase-telomere axis through inducing P300-Sp1 interaction at the −28 to −36 bp region of *hTERT* promoter, and decreased senile cells and elevated expression of *β-Arrestin1* predicted poor prognosis in B-ALL. Collectively, our data shed light on a previously unexpected role of *β-Arrestin1* in the cellular senescence of B-ALL LICs, and showed that *β-Arrestin1*, this protein working with P300-Sp1 is a key regulator for *hTERT* transcription. Combining with our previous reports of the role of *β-Arrestin1* on the self-renewal^5^ and proliferation^4^ of LICs, here we extend the critical role of *β-Arrestin1* on cellular senescence of LICs.

The conventional idea is that tumor cells lost the ability to senescence. Thus, an increasing number of studies clarified that inducible senescence is one of the mechanisms for chemical drug,^7^ and is even responsible for prognosis in leukemia and some solid tumors.^27^ Recently, Collado proposed that tumors might still undergo senescence.^28^ In our study, the senescence was present in leukemic patients and mice, but the percentage of senile cells was less than those in non-leukemic patients and mice, respectively. And our data first proved that the percentage of senile cells may be a novel factor for B-ALL prognosis. The reasons for this are as follows: On one hand, not only senescence could cause tumor cells to be cleared by immune cells, possibly, resulting in efficient tumor regression,^29^ but also senescence could reflect the limit of stemness.^27^ On the other hand, both tumor regression and limited stemness are markers for excellent prognosis of patients with tumors.^30, 31^ Therefore, we deduce that cell senescence is critical for B-ALL prognosis.

We detected the ROS level for stress-induced senescence, hypothesizing that there is a difference between a patient with leukemia and a healthy person, but the effect of *β*-Arrestin1 on ROS was rare in leukemia. Moreover, the senescence-related oncogenes or tumor suppressors, including *P16, P53, P21*, and *P27* were investigated, and the expression of *P16* was significantly different. P16 is involved in premature senescence as a tumor suppressor,^32^ which is also involved in replicative senescence by the P16-RB pathway.^33^ Thus, we explored the role of *β*-Arrestin1 on telomere-related replicative senescence, and found that *β*-Arrestin1 regulated the senescence via the *hTERT*-telomerase-telomere axis. However, the clear role of *P16* gene on this process still need further study.

Abundant evidence has shown that the length of the telomere depended on the activity of telomerase. Regulation of telomerase is multifactorial involving hTERT expression, post-translational protein–protein interactions, and protein phosphorylation.^34^ In our study, we found that the alteration of *hTERT* mRNA expression was positively correlated with the alteration of telomerase activity in the Reh cells' presence or absence of *β-Arrestin1*, so we concentrated on studying the expression of the hTERT gene. The effect of other factors on telomerase needs further investigation.

Many ways for regulating *hTERT* expression, including miRNA,^35^ tumor suppressor or oncogene pathways,^36^ epigenetic mechanisms,^37^ and transcriptional regulation.^38^ *β*-Arrestin1, as a possible oncogene in B-ALL, could be involved in these pathways to regulate the epigenetic and gene transcription. Thus, based on transcriptional regulation of the *hTERT* gene being the major mechanism for cancer-specific activation of telomerase, an activator of transcription was applied to study the possible mechanism for *β*-Arrestin1 regulating hTERT expression. We cannot rule out other possible factors and potential mechanisms, especially miRNA and other epigenetic influence for hTERT expression.

A number of factors have been directly or indirectly identified as regulating the *hTERT* promoter, including cellular transcriptional activators (c-Myc, Sp1, HIF-1, AP2, ER, Ets, etc.) as well as repressors.^14, 39^ Recent research showed, however, that no reasons could clearly account for the cancer specificity of hTERT expression; the chromatin structure has been suggested as an important factor for the binding of transcriptional activators with the *hTERT* promoter.^37^ It has been reported that P300, histone acetyltransferase, mediated by *β*-Arrestin1 (ref. 40 and binding with Sp1 (ref. 20 regulates the gene transcription. Although we demonstrated that *β-Arrestin1* increased the binding of P300 with Sp1, thus promoting *hTERT* transcription in this study, the change of the chromatin structure needs further exploration.

An increasing number of potential drugs targeting the telomere and telomerase have been designed to treat tumors, and most of strategies have been proposed for telomerase inhibition such as BIBR1532 (ref. 41 or telomere uncapping like 6-thio-dG^42^ and Ber8 (ref. 43 at present. In our study, we designed the anti-sense nucleotide for the fragment (−28 to −36 bp) of *hTERT* promoter, and identified the effect. The chemical drug with the anti-sense oligo-DNA for −28 to −36 bp in *hTERT* promoter is promising and needs to further study. Collectively, our results have provided novel insights into the *β-Arrestin1*-mediated regulation of the senescence in leukemic cells, which may benefit for pediatric B-ALL patients’ treatment.

## Materials and methods

### Isolated and identified different cell fractions

We collected the LICs and control cells from BM of B-ALL patients using the magnetic-activated cell sorting (MACS; Miltenyi, Bergisch Gladbach, Germany) system with anti-CD34, anti-CD38, and anti-CD19 antibodies. The purity of the cell fractions was identified by flow cytometry as previous report.^26^

### Senescence-associated beta-galactosidase (SA-*β*-gal) activity assay

SA-*β*-gal activity was measured as mentioned previously with minor modifications. Bone marrow cells from pediatric B-ALL patients or leukemic mice, or Reh cells, were washed with PBS, fixed in 3% formaldehyde for 20 min at room temperature, washed, and incubated with 1 ml staining solution (Beyotime Biotech., Shanghai, China) overnight at 37 °C, 5% CO_2_. We counted the blue-stained cells in at least 200 cells, and calculated the percentage of positive cells.

### TRAP and ELISA

A quantitative measurement of telomerase activity was conducted one of the two ways: either using PCR-based telomeric repeat amplification protocol (TRAP assay) (Roche, Mannheim, Germany) or using the PCR-ELISA (Roche), with the absorbance read at OD450 nm applying an ELISA reader.

### Southern blot

The measurements of terminal restriction fragment (TRF) length in tumor and normal samples were applied using the TeloTAGGG telomere length assay kit (Roche), according to the manufacturer's protocols. The intensity of the hybridization was evaluated by densitometric analysis with Quantity One software (Bio Rad, Hercules, USA) and mean TRF length was estimated according to the formula as described.^44^

### FISH

The slides were processed with the Q-FISH method, as reported previously. In brief, the telomeres were labeled with a Cy3-labeled 5′-CCCTAACCCTAACCCTAA-3′ probe (Fasmac, Atsugi, Japan) and the centromeres were labeled with an FITC-labeled 5′-CTTCGTTGGAAACGGGGT-3′ probe (CENP1: a non-specific centromere probe, Fasmac). Microscopic images were captured with the Image-Pro Plus software package (version 5.0, Media Cybernetics, MD, USA), and analyzed using the public domain NIH image program. We calculated the average fluorescence intensity of telomeres within each nucleus in the FISH images.^45^

### Electrophoretic Mobility Shift Assay (EMSA)

Binding of the Sp1 protein with gene was detected by using LightShift Chemiluminescent EMSA Kit (Thermo, Rockford, USA), according to the manufacturer’s protocol. The Sp1 probe was labeled by biotin and Sp1 consensus oligo was 5′-ATTCGATCGGGGCGGGGCGAGC-3′, 3′-TAAGCTAGCCCCGCCCCGCTCG-5′ (Beyotime Biotech). The supershift was performed with the anti-Sp1 antibody (Abcam, Cambridge, USA).

### Luciferase reporter assay

Luciferase reporter plasmids were kindly provided by Professor Weihui Zhou.^46^ Reh cells were incubated in 10 cm plates (1 × 10^6^ cells per plate) for 24 h, and then transfected with the constructed reporter plasmids (10 *μ*g) and pRL-SV40 (0.01 *μ*g) according to the handbook of effect transfection reagent (Qiagen, Hilden, Germany). Luciferase activity was measured with a dual-luciferase reporter assay system (Promega, Madison, USA) and normalized against the Renilla luciferase activity. All experiments were performed at least three times in each plasmid and represented as the average relative luciferase activity.
