# Supplementary material for: The cellular senescence of leukemia-initiating cells from acute lymphoblastic leukemia is postponed by β-Arrestin1 binding with P300-Sp1 to regulate hTERT transcription
Source: Cell Death Dis. 2017 Apr 20;8(4):e2756–. doi: 10.1038/cddis.2017.164 (PMC5603829; doi:10.1038/cddis.2017.164)

**Material and Methods**

**Clinical patients and samples**

Bone marrow (BM) and peripheral blood (PB) were obtained from newly diagnosed patients who provided informed consent, and the protocol was approved by the Ethics Committee of the Children’s Hospital of Chongqing Medical University. The diagnoses for B-ALL patients were based on the Children’s Cancer and Leukemia Group 2008 study (CCLG-2008). Detailed information about these patients is provided in Supplementary Table S1.

**Animal procedures**

3- to 4-week old NOD/SCID mice were obtained from the Laboratory Animal Center of Chongqing Medical University. All mice were sublethally irradiated with 2cGy in the 3^rd^ Military Medical University(Chongqing, China) and then received one intraperitoneal injection of a 200-µg purified anti-CD122 antibody [1] (kindly provided by Prof. Dengli Hong from Shanghai Jiaotong University) immediately after irradiating. Then, 1×10^4^ different subpopulation cells isolated from B-ALL patients were injected into the mice by tail vein in 24 hours. The mice died or were sacrificed when they exhibited obvious leukemia symptoms. All experimental procedures were performed according to the guidelines established by the animal laboratory committee in our university.

**The telomerase inhibitors (BIBR1532)**

Reh cells were exposed to 100 nM, 300 nM, 500 nM, and 1000 nM BIBR1532 or the equivalent amount of DMSO daily for 48 hours. Each leukemic mouse was intraperitoneally injected with 1nM BIBR1532, every 3 days and 8 times in total.

**Cell culture and Lentivirus infection**

Reh and HEK293T cells (American Type Culture Collection, USA) were cultured and maintained in RPMI 1640 medium and DMEM (Gibco-BRL, USA), respectively. And cells were transfected with vectors or plasmids according to the Effect Transfection Reagent Handbook (Qiagen, Germany). All of the recombined lentiviral vectors of β-Arrestin1 were kindly provided by Prof. Gang Pei [2]. The nucleotide sequences carried in the plasmids are 5’-GGAAGCTCAAGCACGAAGACAA-3’. Gene expression was confirmed by real-time reverse transcription-polymerase chain reaction (RT-PCR) and Western Blot.

**Western Blot**

The whole-cell or nuclear lysates were performed using PAGE electrophoresis, incubated with primary antibodies against β-Arrestin1, Sp1, P300, Lamin B, β-actin, TBP, HIRA, CBX, etc., respectively, followed with their corresponding secondary antibodies, and blotted (Abcam, USA).

**RT-PCR**

Total RNA was isolated from normal donors, B-ALL patients, mice and cell lines using Trizol reagent (Invitrogen, USA) and then retrotranscribed using the PrimeScript^TM^RT reagent Kit with gDNA Eraser (TAKARA, Janpan) according to the manufacturer’s protocol. Real-time reverse transcription-polymerase chain reaction (RT-PCR) was performed using the 7300 Real Time PCR System (Applied Biosystems). The data was described with the value normalized to the mRNA expression of inner control GAPDH according to the 2-∆CT method. The primer sequences are listed in Supplementary Table S2.

**Co-Immunoprecipitation (Co-IP)**

For co-immunoprecipitation (Co-IP) assays, whole cell lysates were first pre-cleared with normal IgG and protein A/G plus conjugated agarose beads (Santa Cruz, USA), and then incubated with anti-Sp1 or anti-P300 (Abcam, USA) under gentle rocking at 4℃ overnight. Protein A/G plus was added, and the mixture was incubated for over 4 hours at 4℃. Following centrifugation, beads were washed with cold PBS and boiled in an SDS-loading buffer. The samples were subjected to SDS-PAGE and Western Blot.

**Immunofluorescence and confocal microscopy**

Smears with Reh cells were fixed in 4% paraformaldehyde PBS at room temperature, washed and permeabilized with methylalcohol, and blocked with 1% BSA in PBS. The cells were then incubated with the indicated primary antibodies overnight at 4°C, and subsequently stained with corresponding secondary antibodies (Abcam, USA), and mounted with glass slides. The signaling was then visualized under a fluorescent microscope (Nicon, Japan). The colocalization of Sp1-P300 or P300-β-Arrestin1 was measured by immunofluorescence and observed through a confocal microscope. Images were shown as a single z-section.

**Statistical analysis**

The significance of the differences in gene expression was determined using Student’s *t*-test. Differences of *p*<0.05 were considered significant. The *p* value for survival analysis was calculated by Log-Rank. All statistical analysis was performed using the GraphPad Prism (Version 5.0) and the SPSS (Version 17.0) software.

**Reference**

1 [Duan CW](http://www.ncbi.nlm.nih.gov/pubmed/?term=Duan%20CW%5BAuthor%5D&cauthor=true&cauthor_uid=24937459), [Shi J](http://www.ncbi.nlm.nih.gov/pubmed/?term=Shi%20J%5BAuthor%5D&cauthor=true&cauthor_uid=24937459), [Chen J](http://www.ncbi.nlm.nih.gov/pubmed/?term=Chen%20J%5BAuthor%5D&cauthor=true&cauthor_uid=24937459), Wang B, Yu YH, Qin X, Zhou XC, Cai YJ, Li ZQ, Zhang F, Yin MZ, Tao Y, Mi JQ, Li LH, Enver T, Chen GQ, Hong DL. Leukemia propagating cells rebuild an evolving niche in response to therapy. Cancer Cell. 2014; 25(6): 778-93.

2 [Li Y](http://www.ncbi.nlm.nih.gov/pubmed/?term=Li%20Y%5BAuthor%5D&cauthor=true&cauthor_uid=19399231), [Liu X](http://www.ncbi.nlm.nih.gov/pubmed/?term=Liu%20X%5BAuthor%5D&cauthor=true&cauthor_uid=19399231), [Liu C](http://www.ncbi.nlm.nih.gov/pubmed/?term=Liu%20C%5BAuthor%5D&cauthor=true&cauthor_uid=19399231), Liu C, Kang JH, Yang JY, Pei G, Wu CF. Improvement of morphine-mediated analgesia by inhibition of beta-arrestin2 expression in mice periaqueductal gray matter. Int J Mol Sci. 2009; 10(3): 954-63.

**Supplementary Table S1. The Clinical information of patients**

| **Patient**  **No.** | **Age**  **(Y)** | **Gender** | **WBC**  **(×10^9^/L)** | **BM blast cells (%)** | **Morphology** | **Immunology** | **Fusion Gene** |
| --- | --- | --- | --- | --- | --- | --- | --- |
| 1 | 3 3/12 | F | 41.24 | 97.5 | L2 | Pre-B | NA |
| 2 | 2 7/12 | M | 3.17 | 80 | L2 | Pre-B | NA |
| 3 | 5 4/12 | F | 2.71 | 80.5 | L1 | C-ALL | NA |
| 4 | 2 1/12 | M | 4.18 | 91 | L1 | C-ALL | TEL/AML1 |
| 5 | 8 3/12 | M | 4.4 | 88.5 | L1 | C-ALL | NA |
| 6 | 5 3/12 | M | 26.78 | 81 | L1 | Pre-B | E2A/PBX |
| 7 | 5/12 | M | 11.66 | 86.5 | L1 | C-ALL | NA |
| 8 | 7 4/12 | M | 127.42 | 95 | L2 | Pre-B | NA |
| 9 | 9 3/12 | M | 78.21 | 70 | L1 | C-ALL | BCR/ABL |
| 10 | 7 2/12 | F | 3.05 | 87 | L2 | C-ALL | NA |
| 11 | 4 5/12 | M | 4.39 | 92.5 | L1 | C-ALL | NA |
| 12 | 2 6/12 | F | 140.48 | 67.3 | L2 | Pre-B | E2A/PBX1 |
| 13 | 3 2/12 | F | 58 | 64.1 | L2 | C-ALL | NA |
| 14 | 2 6/12 | F | 122.32 | 94 | L2 | C-ALL | NA |
| 15 | 3 8/12 | M | 2.62 | 48 | L3 | C-ALL | NA |
| 16 | 13 9/12 | M | 23.19 | 95.5 | L2 | C-ALL | NA |
| 17 | 8 5/12 | M | 130.65 | 95 | L1 | C-ALL | NA |
| 18 | 4 7/12 | F | 56.43 | 88 | L2 | Pre-B | NA |
| 19 | 2 10/12 | M | 35.43 | 90 | L2 | C-ALL | NA |
| 20 | 2 9/12 | M | 49.03 | 95 | L2 | C-ALL | NA |
| 21 | 1 5/12 | F | 8.72 | NA | NL(IM) | NA | NA |
| 22 | 1 11/12 | M | 7.35 | NA | NL(IM) | NA | NA |
| 23 | 1 5/12 | F | 5.06 | 4 | NL(IM) | NA | NA |
| 24 | 3 9/12 | M | 9.02 | 0.5 | NL(ITP) | NA | NA |
| 25 | 3 2/12 | M | 13.17 | NA | NL(G6PD) | NA | NA |
| 26 | 7 3/12 | M | 6.0 | 0.5 | NL(ITP) | NA | NA |
| 27 | 4 6/12 | M | 10.17 | 3.5 | NL(ITP) | NA | NA |
| 28 | 2 8/12 | F | 17.09 | 0.5 | NL(G6PD) | NA | NA |
| 29 | 6 9/12 | F | 7.46 | 1 | NL(ITP) | NA | NA |
| 30 | 2 1/12 | F | 4.57 | NA | NL(ITP) | NA | NA |
| 31 | 5 1/12 | M | 13.79 | 1.5 | NL(ITP) | NA | NA |
| 32 | 1 6/12 | F | 10.36 | 0.5 | NL(ITP) | NA | NA |
| 33 | 5 11/12 | F | 8.36 | 2 | NL(ITP) | NA | NA |
| 34 | 1 6/12 | M | 10.54 | 0.5 | NL(IDA) | NA | NA |
| 35 | 4 4/12 | F | 12.26 | 0.5 | NL(ITP) | NA | NA |
| 36 | 2 3/12 | F | 10.2 | 1 | NL(ITP) | NA | NA |
| 37 | 1 3/12 | M | 6.85 | 0.5 | NL(IDA) | NA | NA |
| 38 | 6/12 | M | 7.25 | 0.5 | NL(IDA) | NA | NA |
| 39 | 2 5/12 | F | 4.59 | 0.5 | NL(ITP) | NA | NA |
| 40 | 5 7/12 | M | 5.68 | 1 | NL(IM) | NA | NA |

Abbreviation: No. , number; Y: years; WBC, white blood cell; BM, bone marrow; F, female; M, male; Pre-B, precursor B-ALL; C-ALL, common B-ALL; NL, non-leukemic patients; IM, infectious mononucleosis; ITP, idiopathic thrombocytopenic purpura; G6PD, Glucose-6-Phosphate Dehydrogenase deficiency; IDA, Iron deficiency anemia; NA, Not Available.

**Supplementary Table S2: The sequence for primers and sh-RNAs**

| **Gene Name** | **Primer Sequence** |
| --- | --- |
| β-arrestin1 | F：GACAAAGGGACCCGAGTGTT  R：GCAGGTCAGCGTCACATAGA |
| P16 | F：GGAAATTGGAAACTGGAA  R：TCTGAGCTTTGGAAGC |
| P53 | F：GCCACCTGAGTCTGCAATGA  R：GCAGCCCAATCCTAGAAGCA |
| P21 | F：TCTTCTGTTTCAGCCACAGGC  R：TGTCAGGCTGGTCTGCCTC |
| P27 | F：TAAGGAAGCGACCTGCAACC  R：GCAGAAGTTGGCCCCATAGT |
| hTERT | F：TGTTTCTGGATTTGCAGGTG  R：GTTCTTGGCTTTCAGGATGG |
| Sp1_1_ | F：CGGGGTACCTTTCCGCGGCCCCGCCCTCT  R：CCCAAGCTTTGCGCAGCAGGACGCAGCG |
| Sp1_1-2_ | F：CGGGGTACCTCCCAGCCCCTCCCCTTC  R：CCC AAGCTTTGCGCAGCAGGACGCAGC |
| Sp1_1-5_ | F：ATTGGTACCCCTGCCCCTTCACCTTCCA  R：ATAAAGCTTGCGCTGCCTGAAACTCGC |
| Sp1_5_+E-box | F：CGGGGTACCTGGATTCGCGGGCACAGAC  R：CCCAAGCTTAGGAGGCGGAGCTGGAAGGT |
| NSp1+E-box | F：ATTGGTACCTGGATTCGCGGGCACAGA  R：ATAAAGCTTGCGGAGCTGGAAGGTGAAGG |
| IL2Rgc | F：GTGGGTAGCCAGCTCTTCAG  R：CCTGGAGCTGGACAACAAAT |
| P300 | F：CAGGGCCTAACATGGGACAG  R：CGAGGCATCATCTGGTTTGG |
| sh-RNA-P300 477 | T：TCACTTTATGGAAGAGTTA |
| sh-RNA-P300 478 | T：AGCCTCAAACTACAATAAA |
| sh-RNA-P300 479 | T：CTTCACAATTCCGAGACAT |
| sh-RNA-P300 480 | T：CGGTGAACTCTCCTATAAT |

Abbreviation: F, forward primer; R, reverse primer; T, target sequence; SP1_1,_ the core fragment of hTERT gene located at -18bp～+15bp; SP1_1-2,_ the core fragment of hTERT gene located at -37bp～+15bp; SP1_5_+E-box_,_ the core fragment of hTERT gene located at -202bp～-100bp; Sp1_1-5,_ the core fragment of hTERT gene located at -130bp～-2bp; NSp1+E-box_,_ the core fragment of hTERT gene located at -202bp～-110bp; IL-2 R γ, IL-2 receptor γ chain.

**Supplementary Figure S1** The correlation of senile cells with the expression of β-Arrestin1 mRNA in four fractions from LICs

CD34+CD38-CD19+ cells, CD34+CD38+CD19+ cells, CD34-CD38+CD19+ cells, and CD34-CD38-CD19+ cells were isolated from newly diagnosed B-ALL patients by MACS. (A) The images of identifying by FACS were shown. (B) The representative images of senile cells in four fractions by SA-β-gal staining. (C) The statistic of senile cells in four fractions. The correlation of the percentage of senile cells with the expression of β-Arrestin1 mRNA in CD34+CD38-CD19+ cells (D), CD34+CD38+CD19+ cells (E), CD34-CD38+CD19+ cells (F), and CD34-CD38-CD19+ cells (G). Scale bar, 5µm.

**Supplementary Figure S2** Identification of mice models derived from B-ALL patients and depleted β-Arrestin1 in leukemic cells and mice

1. CD34+CD38-CD19+ fractions from B-ALL patients were isolated by MACS and identified by FACS. (B) The scheme of the leukemic NSG model. (C) The IL-2Rgc receptor was measured in the genomic DNA of W/M mice by PCR and gel electrophoresis. (D) The loss of β-Arrestin1 in NSG mice was detected by Western Blot (top) and relative RT-PCR (bottom). (E) The propagation of human leukemic cells in NSG mice was measured through staining the PB smear, BM smear by Wright’s stain, and leukocyte infiltration in spleen and liver by H&E stain. M, mice injected with anti-CD122; W, mice injected without anti-CD122; Scale bar, 5µm.

**Supplementary Figure 3** The correlation of senile cells with the expression of β-Arrestin1 mRNA in BM cell from leukemic mice

Mixed CD34+CD38-CD19+ cells from BM of patients #1, #2, and #3 were injected into the 10 NSG mice. Meanwhile, CD34+CD38-CD19+ cells from non-leukemic patients was also injected into another 10 NSG mice. BM and PB cells were collected, detecting the human B-ALL cells in leukemic NSG mice. Smears were prepared. RNA and protein were purified from PB cells. (A)The β-Arrestin1 mRNA was detected by Western Blot (top) and real-time RT-PCR (bottom); (B) The expression of β-Arrestin1 was detected by i[mmunofluorescence](javascript:void(0);) staining; (C) The role of β-Arrestin1 on predicting the prognosis of leukemic mice; (D) Senescence was detected in PB cells of leukemic mice by β-gal staining. (E) The effect of senescence on the prognosis of leukemic mice; (F) The correlation of the expression of β- arrestin1 with senescence in leukemic mice. Ctrl, non-leukemic mice; B-ALL, B acute lymphoblastic leukemia; n, number; GAP, inner control GAPDH gene.

**Supplementary Figure S4** Intracellular ROS levels in different B-ALL cells

Reh cells (R-Scram, R-Sβ1) and bone marrow cells from leukemic mice (M-Scram, M-Sβ1) were loaded with DCFH and observed by fluorescent microscope. Representative images in Reh (A) and BM cells (C) were obtained. Each trace represents the response of one cell. Three independent experiments were performed and normalized to the value in control groups and the statistical graph in 100 cells in Reh (B) and BM cells (D) respectively. Scale bar, 5µm.

**Supplementary Figure S5** Different concentrations of BIBR1532 treated on Reh cells

Reh cells were seeded in 24-well plates at a density of 5x10^4^ cells per well. After 24 hours, the cells were treated with different concentrations of BIBR1532 for the indicated time; then the culture media was discarded and cells were harvested. We measured the cell growth curve of Reh cells treated with BIBR1532 from Day 1 to Day 5 (A), the hTERT mRNA expression by RT-PCR and presented as relative fold in Reh cells (B), and the hTERT mRNA expression by RT-PCR and presented as relative fold in different Reh cells with 3µmol/L BIBR1532 (C).

**Supplementary Figure S6** Different binding sites for Sp1 in the promoter

(A) The proximal core sequence of the hTERT promoter and the binding sites for transcription factor Sp1. (B) The expression of Sp1 protein in Reh cells by Western Blot. (C) The scheme of different clone fragments of the hTERT promoter into PGL3-basic vector. (D) The expression of β-Arrestin1 in Reh cells by RT-PCR and Western Blot.

**Supplementary Figure S7 Loss of β-Arrestin1 inhibits hTERT expression, decreases telomerase activity, and shortens telomere length in leukemic mice**

DNA was extracted from tissues form M-Siβ1 and M-Scram mice. The length of telomere was detected by Southern blot. Calculating telomere length was performed with optical density analysis, according to the kit’s protocol. The average length of telomere (TRF) in different groups was calculated and compared with the control group. The representative images of the Southern blot (A) and the TRF (B) for B-ALL-LICs-derived mice. The length of telomere was also detected by FISH in BM smear. The representative images of FISH (C); after calculating the fluorescence intensity of telomeres, the value was quantified relative to the level of Scram group without BIBR1532 and are presented as the relative intensity of fluorescence (D). The activity of telomerase was measured by PCR-TRAP (E). The hTERT mRNA expression fold was detected by RT-PCR (F). All *p* values were calculated by t-test. **p*<0.05, ***p*<0.001; B, the inhibitor of telomerase (BIBR1532); M, marker; P, positive control; TE, the activity of telomerase; TRF, the average length of telomere. Scale bar, 10 µm.

**Supplementary Figure S8 Statistics for Southern Blot, FISH and Senile cell.**

R-Siβ1 cells and R-Scram cells were transfected by anti-sense oligonucleotide of Sp1_2_ (-28bp~-36bp). The length of telomere is detected by Southern Blot and FISH, statistics is presented in (A) and (B) respectively. The senile cell is stained by SA-β-gal staining, and the percentage of senile cell is presented in (C).


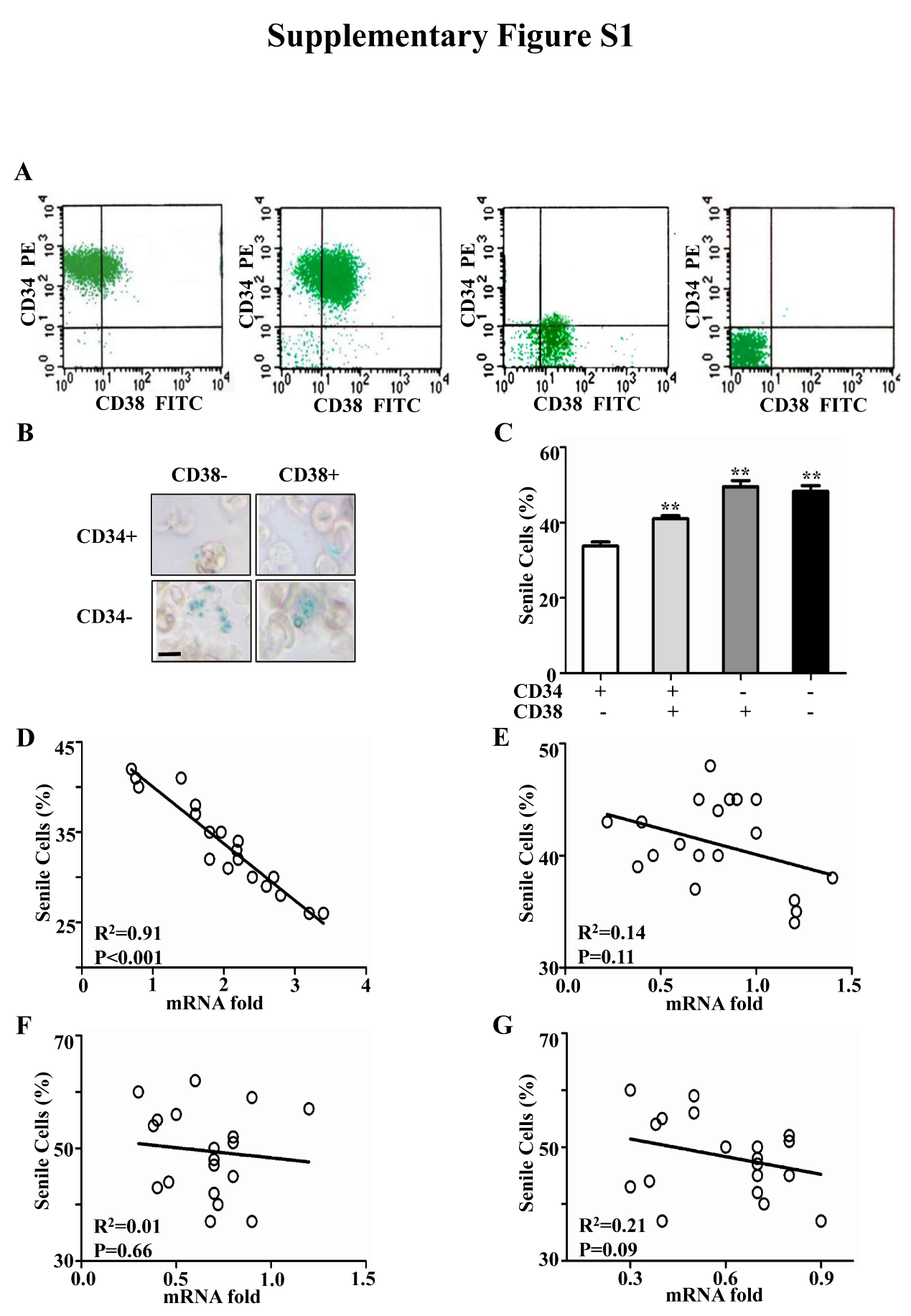


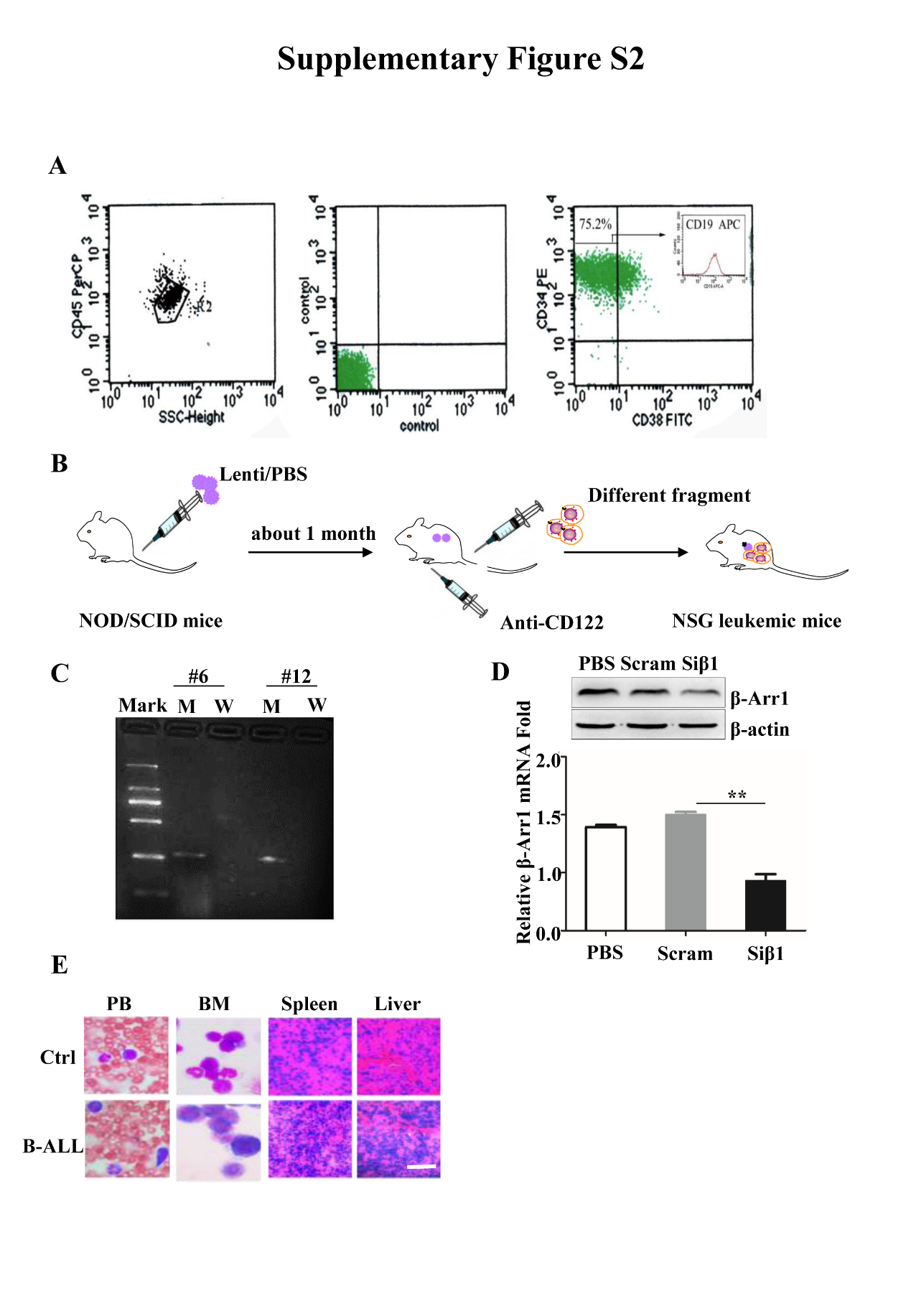


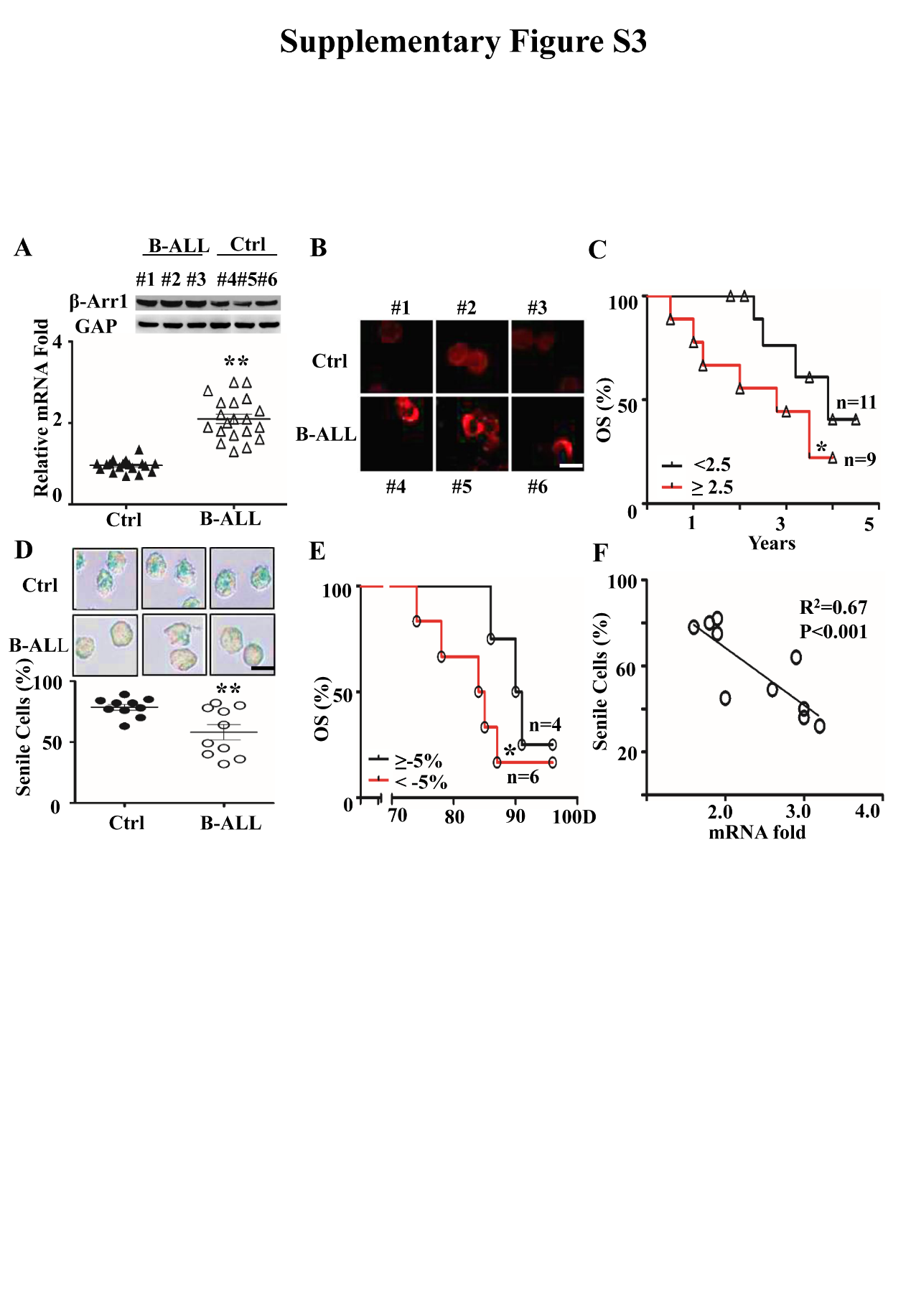


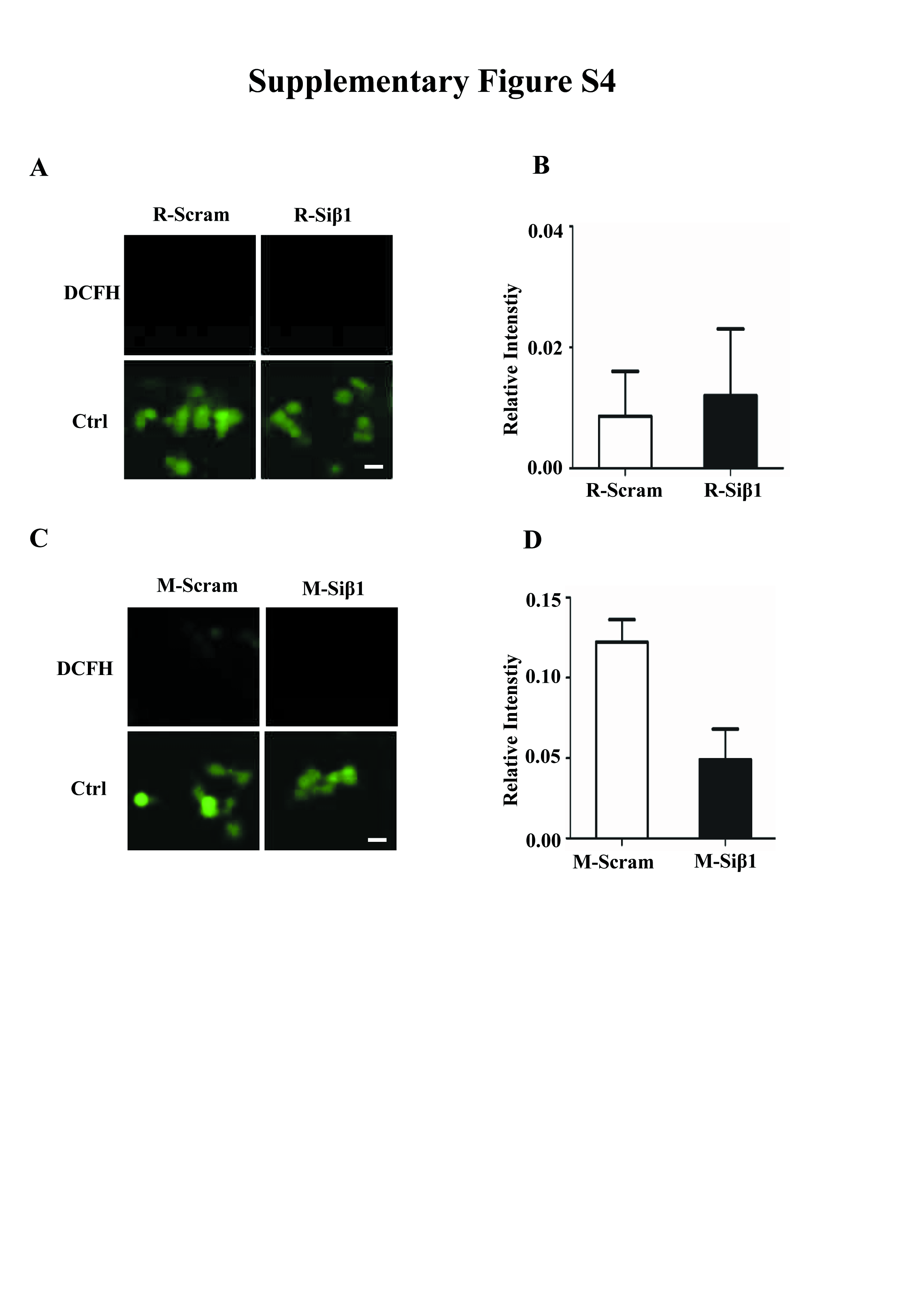


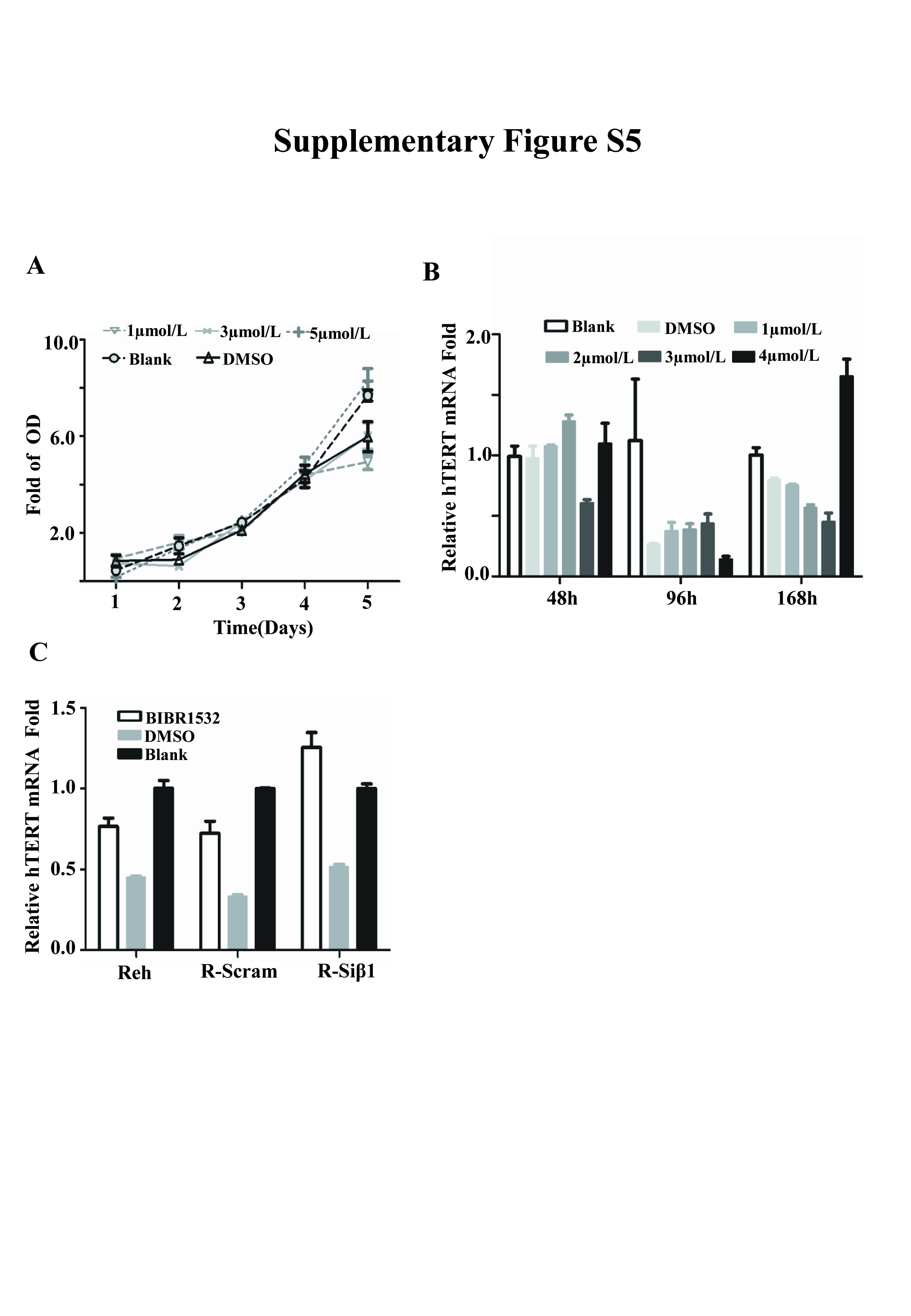


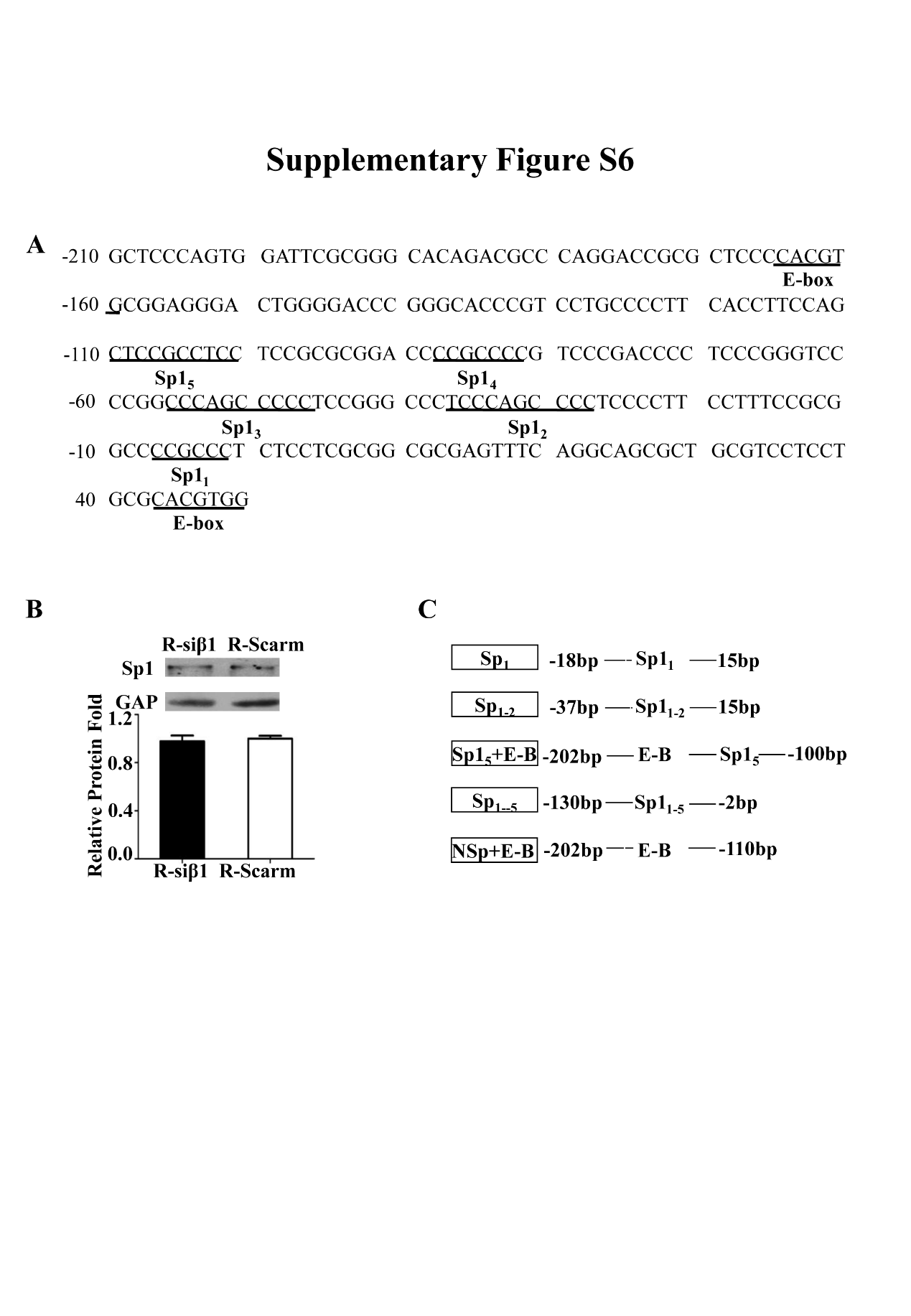


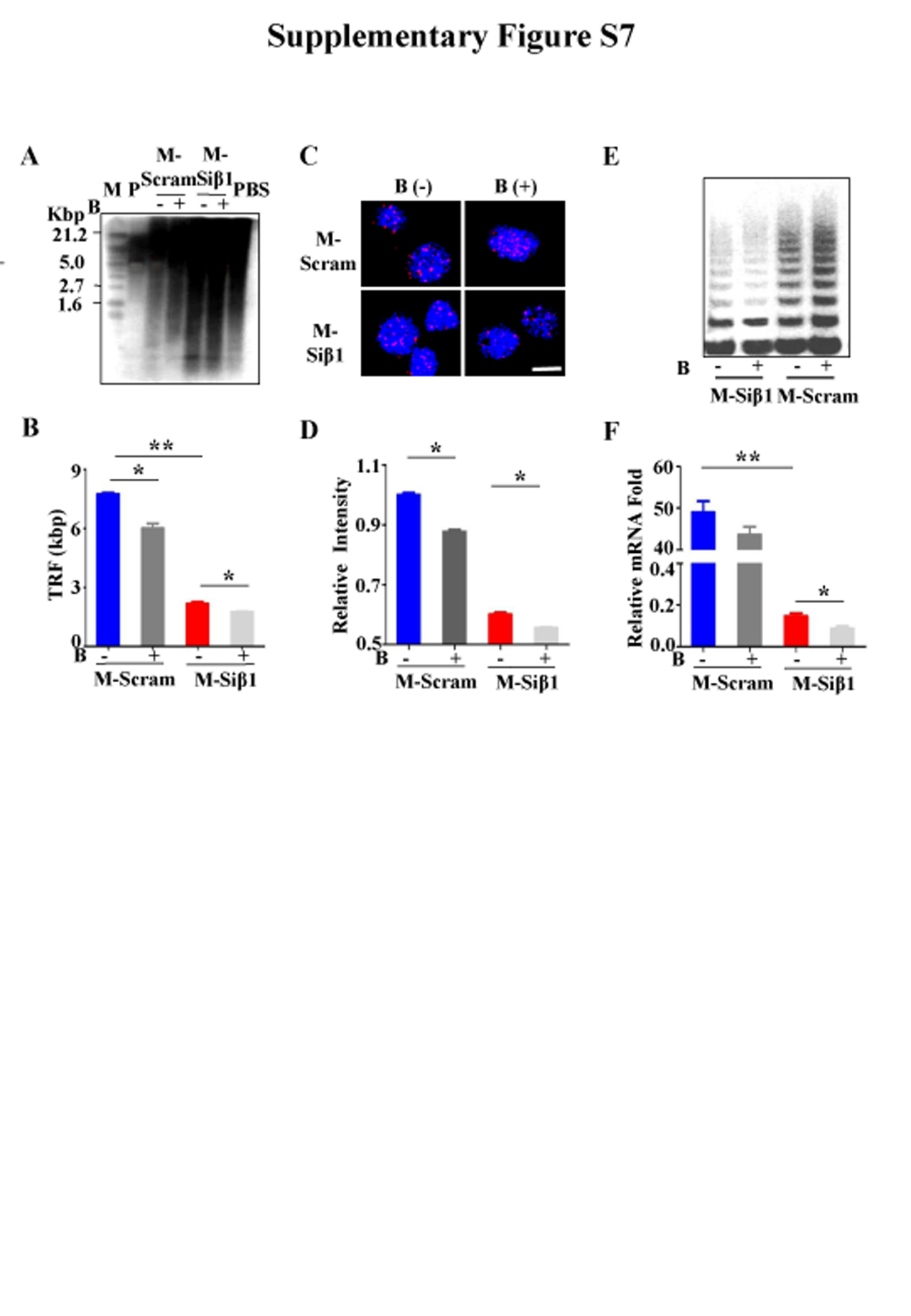


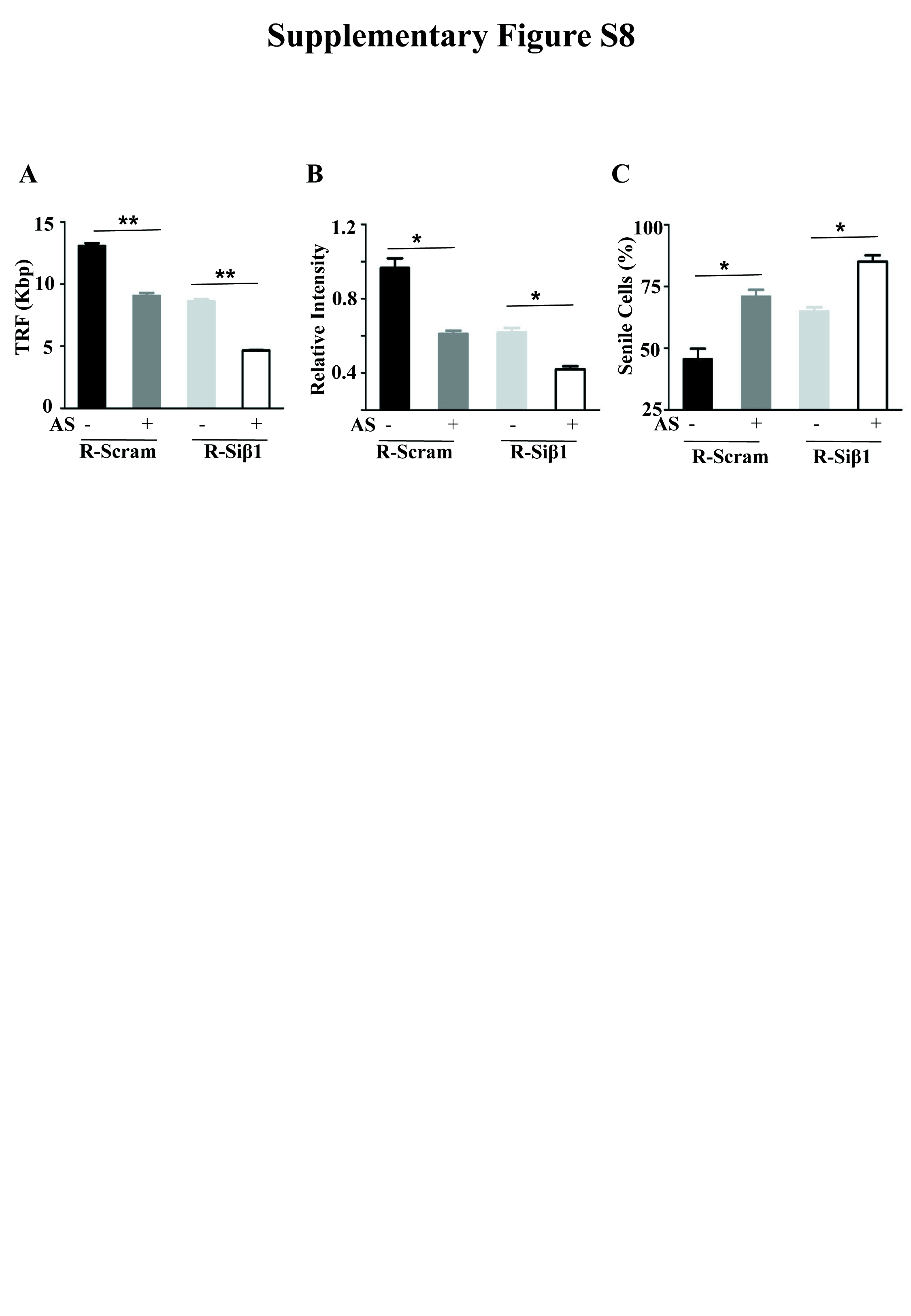

Supplement: Supplementary Information [file cddis2017164x1.docx]
